# Effect of Industrial Pollution in Puchuncaví Valley on the Medicinal Properties of *Senecio fistulosus* Poepp. ex Les (Asteraceae): Content of Phytoconstituents and Their Antioxidant and Cytotoxic Activities

**DOI:** 10.3390/molecules28207038

**Published:** 2023-10-12

**Authors:** Manuel Martínez-Lobos, Estela Tapia-Venegas, Paula Celis-Plá, Joan Villena, Carlos Jara-Gutiérrez, Alexandra Lobos Pessini, Alejandro Madrid-Villegas

**Affiliations:** 1 Programa de Doctorado Interdisciplinario en Ciencias Ambientales, Facultad de Ciencias Naturales y Exactas, Universidad de Playa Ancha, Valparaíso 2360002, Chile; 2 Laboratorio de Productos Naturales y Síntesis Orgánica, Universidad de Playa Ancha, Av. Leopoldo Carvallo 270, Valparaíso 2360002, Chile; alejandro.madrid@upla.cl; 3 Departamento de Ciencias de la Ingeniería para la Sostenibilidad, Facultad de Ingeniería, Universidad de Playa Ancha, Valparaíso 2360002, Chile; estela.tapia@upla.cl; 4 Laboratorio de Bioprocesos, HUB Ambiental UPLA, Universidad de Playa Ancha, Valparaíso 2360004, Chile; 5 Laboratorio de Investigación Ambiental Acuática (LACER), HUB Ambiental UPLA, Universidad de Playa Ancha, Valparaíso 2360002, Chile; paulacelispla@upla.cl; 6 Departamento de Ciencias Naturales y Geografía, Facultad de Ciencias Naturales y Exactas, Universidad de Playa Ancha, Valparaíso 2360002, Chile; 7 Laboratorio de Bioensayos, Universidad de Valparaíso, Angamos 655, Viña del Mar 2340064, Chile; juan.villena@uv.cl (J.V.); carlos.jara@uv.cl (C.J.-G.); 8 Laboratorio Silob Chile, Javiera Carrera 839, Valparaíso 2390410, Chile; alobos@silobchile.cl

**Keywords:** abiotic stress, phytoconstituents, medicinal plants, *Senecio fistulosus*, hexadecanoic acid, (Z,Z,Z)-9,12,15-octadecatrienoic acid

## Abstract

*Senecio fistulosus*, an endemic plant in Chile, is highly regarded for its medicinal properties and is popular in alternative medicine. It thrives even in polluted areas, like Puchuncaví Valley, Chile. Therefore, the study aimed to assess the impact of industrial pollution in Puchuncaví Valley, Chile, on the phytoconstituent content, as well as the antioxidant and cytotoxic activities, of *S. fistulosus*. Phenols, flavonoids, and anthraquinones content were measured, alongside the assessment of antioxidant activities. Additionally, a GC-MS analysis was conducted to profile the phytoconstituents, while the cytotoxic potential was evaluated in HT-29 and MCF-7 and cell line non-tumorigenic MCF-10. The Wild sample exhibited a greater concentration of phytoconstituents (0 to 169.48 mg·L^−1^) compared to the Commercial control (0 to 95.38 mg·L^−1^), directly correlating with its antioxidant activity. While the Wild species showed cytotoxic activity, the Commercial control demonstrated cytotoxic effects on MCF-10 and MCF-7. Noteworthy compounds identified were hexadecanoic acid (12.76 to 19.57% relative area) and (Z,Z,Z)-9,12,15-octadecatrienoic acid (18.36% relative area), with anticancer properties. In conclusion, the abiotic stress experienced by *S. fistulosus* led to higher phytoconstituent content and improved antioxidant activity when contrasted with the Commercial control. The Commercial species showed increased cytotoxic activity against both tumorigenic and non-tumorigenic cell lines.

## 1. Introduction

The medicinal properties of plants can be attributed to the presence of various types of secondary metabolites (or phytoconstituents), such as terpenoids, phenols, flavonoids, and anthraquinones, among others [1,2,3]. These molecules provide plants with antioxidant, anticancer, antimicrobial, antifungal activities, and other beneficial activities [4,5,6,7,8], which can be used to treat different diseases and/or conditions [9]. Plant families that are widely distributed across various environmental conditions or are subjected to biotic and/or abiotic stress are expected to have a diverse range of phytoconstituents. Adverse environmental conditions, such as the presence of pollutants, like heavy metals, can vary the formation of these compounds, and can also alter their content [10,11]. This highlights the potential for plants to be a source of novel bioactive compounds that could be used in pharmaceuticals and other applications [12].

The Asteraceae family comprises a vast number of species, reaching up to 25,037 distributed worldwide, except for the Antarctic continent and Greenland [13,14,15,16]. Therefore, this family presents high habitat diversity due to the longitudinal and altitudinal gradient, making it a great source of phytoconstituents [17,18,19,20]. In turn, its *Senecio* genus is one of the most diverse among angiosperms [21] (flowering plants). In Chile, there are around 220 native species of this genus, including 103 endemic species, resulting in a 46% endemism rate [21]. This genus is subjected to various environmental conditions that make it an excellent reservoir of phytoconstituents. Hence, *Senecio* genus possess different medicinal properties, such as anti-inflammatory, antimicrobial, antioxidant, antibacterial, antiviral, antitumor, antiemetic, vasodilator, and analgesic effects. Some of these properties have been useful in treating bronchial asthma, wounds, and other ailments [6,22,23,24,25,26].

*Senecio fistulosus* is a perennial herb commonly known as Hualtata or cow’s tongue. It is a perennial herb, robust, with thick rhizomes and large leaves that are arranged in a rosette and can reach a length of 30 to 50 cm. Its stem can grow up in height and ends in a yellow inflorescence. This plant is typically found in the humid areas of central–southern Chile and in the Valparaíso region and is commonly found near wetlands or watercourses, as is the case in the Puchuncaví Valley [27]. This region is characterized, among other factors, by the presence of a large industrial complex located between the municipalities of Quintero and Puchuncaví. This area has been declared a saturated zone for atmospheric pollutants, including sulfur dioxide (SO_2_), respirable particles (PMs), and others. In addition, due to atmospheric depositions, it is common to find elevated concentrations of heavy metals, such as copper (Cu); lead (Pb); zinc (Zn), cadmium (Cd); and metalloids, such as arsenic (As), in the soil and watercourses within this area [28,29]. 

The literature has identified sesquiterpenes with low amounts of pyrrolizidine alkaloids in *S. fistulosus* [30]. Thus, the most frequent chemical groups found in the non-alkaloid fraction are sesquiterpenes of the eremophyllane, furanoeremophyllane, and eremophyllanolide types. Specifically, two of the 9-oxo-furanoeremophyllane type, one of the eremophylanolide type (1*β*,10*β*-epoxy-6-acetoxy-8*α*-hydroxy-eremophyll-7(11)-en-8*β*,12-olide), and a maaliol derivative were isolated. In addition, pyrrolizidinic alkaloids were also isolated [30]. This type of phytoconstituent would have pharmacological properties on the cardiac muscle, highlighting the potential to be antifibrillant. The genus to which it belongs is characterized by possessing a great variety of pyrrolizidinic alkaloids [30,31]. For this reason, *S. fistulosus* is considered by the Chilean Ministry of Health (Minsal) as a medicinal plant, used for heart disease, bloating, and stomach upset, and is frequently marketed in alternative pharmacies [27,31]. Therefore, it is expected that the species obtained from the Puchuncaví valley has a higher content of phytoconstituents, which would lead to higher antioxidant and cytotoxic activity, considering the commercialized species as a positive control.

The aim of this study was to evaluate the effect of industrial pollution in the Puchuncaví valley on the medicinal applications of *S. fistulosus*, focusing on the phytoconstituent content and its antioxidant and anticarcinogenic activities. 

## 2. Results

### 2.1. Percentage Yield of Extracts

The percentage of yield obtained from the extracts of the species *S. fistulosus* is presented in Table 1, ranged from 0.35 and 9.24%, where the ethanolic (E) extract stands out as the one with the best yield and the ethyl acetate (A) extract as the one with the lowest yield in both types of samples. 

### 2.2. Photosynthetic Capacity

The photosynthetic capacity of *S. fistulosus* was determined only in the Wild species, yielding the results shown in Table 2, where the effective yield (Yield II) was measured at an irradiance of 1970 µmol photon m^−2^ s^−1^ at 10:00 am. Also, the maximum yield (*Fv/Fm*) obtained after 15 min of shade is observed. In addition, we can find the in situ electron transport rate (ETR in situ) that establishes the productivity of the species under the aforementioned conditions. Finally, the absorptance value is observed, which refers to the amount of light that passes through photosystem two. These data show that the species does not present photoinhibition; therefore, it is possible to point out that the species has a good state of health.

### 2.3. Phytoconstituents Content

Table 3 shows the content of phenols, flavonoids, and anthraquinones for both Wild and Commercial species obtained in the UV-Vis spectrophotometric assays. According to the statistical analysis, significant differences (*p* < 0.05) were observed in these values.

Based on the data presented in Table 3, the concentration of phytoconstituents analyzed (phenols, flavonoids, and anthraquinones concentrations) varied from 0 to 169.48 mg·L^−1^. Medium-polarity extracts (D and A) had the highest content of these metabolites, ranging from 3.60 to 169.48 mg·L^−1^, showing differences with the hexane and ethanol extracts, which ranged from 0 to 70.63 mg·L^−1^.

It is worth noting that extracts from Wild species had higher contents of phytoconstituents, ranging from 0 to 169.48 mg·L^−1^, in contrast to the Commercial species, which varied from 0 to 95.38 mg·L^−1^. The major phytoconstituent found was total phenols among the Wild and Commercial extracts, ranging from 27.53 to 169.48 mg·L^−1^ and 40.03 to 95.39 mg·L^−1^, respectively. The second major phytoconstituent found was total flavonoids. These varied between Wild and Commercial extracts from 4.17 to 15.42 mg·L^−1^ and 1.08 to 13.10 mg·L^−1^, respectively. Finally, anthraquinones varied from 0 to 31.08 mg·L^−1^ and from 0 to 9.63 mg·L^−1^ for Wild and Commercial extracts, respectively. Therefore, it is clear from the results that the Wild species had a higher content of phytoconstituents than the Commercial species and that the medium-polarity solvents had a higher content of phytoconstituents. 

### 2.4. Antioxidant Activity

Table 4 shows the antioxidant activity of different extracts of *S. fistulosus* as determined by three antioxidant assays, DPPH, FRAP, and TRAP. According to DPPH assays, a higher IC_50_ value indicates lower antioxidant activity. The results show that all DPPH assays measured on the Wild-type and Commercial extracts showed significant differences from the positive controls (Trolox and Gallic acid), presenting values of minimum inhibitory concentration (IC_50_) higher than these. However, the medium polarity (D and A) from the Wild-type sample showed values that were closer to those of the positive control (Trolox and Gallic acid). At the same time, extracts D, A, and E from Wild samples had a lower minimum inhibitory concentration (IC_50_) (1.89 to 3.29 mg/kg) compared to the stressed samples (4.24 to 9.2 IC_50_).

In the case of the FRAP assay, it is observed that extracts H, D, and A of the Wild-type species show higher activity (0.12 to 0.54 TEAC mM) compared to the Commercial species (0.08 to 0.17 TEAC mM), particularly, the medium-polarity (D and A) extracts, which showed values of 0.6 and 0.54 TEAC mM, respectively, for Wild-type and 0.17 and 0.09 TEAC mM, respectively, for Commercial species. In turn, the values obtained in the Wild-type H extract (0.24 TEAC mM) present statistically significant differences compared to the Commercial species (0.08 TEAC mM). The same trend is observed in the TRAP assay, where extracts D, A, and E from the Wild species showed higher antioxidant activity (0.016 TEAC mM in all cases) than Commercial species (0.0032 to 0.0084 TEAC mM), except for extract H. These results suggest that the Wild-type *S. fistulosus* extracts have higher antioxidant activity than the Commercial samples. 

### 2.5. Correlation between Phytoconstituents Content and Antioxidant Activity

Table 5 presents the level of Spearman’s correlation between the content of phytoconstituents and the antioxidant activity of the extracts being studied. The Spearman correlation evaluates the degree of correspondence between phytoconstituents (measured in terms of phenols, anthraquinones, and flavonoids) and antioxidant activity (measured through various DPPH, FRAP, and TRAP assays). In the case of all variables, the closer it is to 1, the higher the degree of correspondence. However, in the case of DPPH, the correlation values are negative because as the antioxidant activity increases, the IC**_50_** value decreases, and therefore, it should be close to −1. The results indicate that there is a high correlation between phenol content and FRAP (r > 0.8 and *p* < 0.05) and TRAP activity (r > 0.7 and *p* < 0.05). Additionally, flavonoids (r > 0.7 and *p* < 0.05) and anthraquinones (r > 0.7 and *p* < 0.05) show the highest correlation with DPPH stable free radical scavenging activity. 

### 2.6. Analysis of Phytoconstituents by GC-MS 

The chemical composition of extracts D and A of Wild and Commercial *S. fistulosus* was determined by GC-MS analysis. GC-MS chromatograms are shown in Figure S1 of the Supplementary Material. The relative area of the compounds detected in dichloromethane (D) and ethyl acetate (A) extracts is shown in Table 6. 

**Figure 1:**
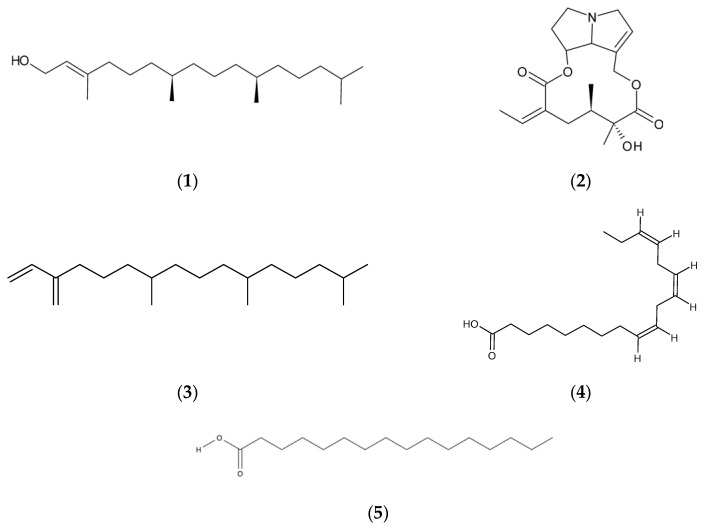
Structure of 5 major compounds identified in Wild and Commercial species in the species *S. fistulosus*, where (**1**) corresponds to Phytol, (**2**) to Senecionine, (**3**) to Neophytadiene, (**4**) to (Z,Z,Z) 9,12,15-Octadecatrienoic acid, and (**5**) to n-Hexadecanoic acid.

The extracts consisted mainly of organic compounds of 6 to 20 carbons, including compounds classified as alkaloids, pyrrolizidine alkaloids, diterpenes, aromatics, and saturated and unsaturated fatty acids; these are listed in Table 6. Extracts D and A of Wild *S. fistulosus* showed a similar chemical composition. As for the D and A extracts of Commercial *S. fistulosus*, they showed a chemical composition different from each other, and different from the composition of the Wild-type species. The five compounds with the highest relative area of all samples are shown in Figure 1.

The Wild sample showed between seven and six metabolites (known and unknown) in both extracts, with a percentage area ranging from 42.79% to 1.95%. The major ones (over 10%) are Phytol (1) (42.79 for D and 5.43% for A), Senecionine (2) (40.02% for D and 15.43 for A), Neophytadiene (3) (20.23% for D and 10.93% for A in Wild, 4.31 for D in Commercial), and Indolizine (12.18% for D and 6.33 for A), which dominated the D and A solvent profile (Figure 1 structures). However, there is a potentially significant compound present in a small proportion (less than 10%): the pyrrolizidine alkaloid Platyphylline. It was identified in the Wild-type species, with a presence of less than 10% (6.44 for D and 1.95 for A). On the other hand, the diacetone alcohol 2-Pentanone. 4-hydroxy-4-methyl- is found in both Wild-type and Commercial species (13.71 for D and 11.60 for A in Wild-type, 2.62 and 2.46 for D and A extracts of the Commercial sample).

When observing the gas chromatogram of the Commercial species, a higher content of compounds (14 and 15 metabolites of known and unknown, respectively) was observed, with a percentage area ranging from 33.10% to 0.59%, of which only Neophytadiene and 2-Pentanone. 4-hydroxy-4-methyl-, the majority, were repeated in the Wild sample, in both cases with a relative area of less than 10% in the Commercial sample.

On the other hand, the metabolites that stand out in the gas chromatogram of the Commercial sample, with a percentage of relative area higher than 10%, in both solvents are (Z,Z,Z) 9,12,15-Octadecatrienoic acid (4) (19.52% for D and 18.36% for A) and n-Hexadecanoic acid (5) (15.72% for D and 12.76% for A) (Structures in Figure 1).

Table 6 represents the relative amounts of compounds detected in dichloromethane (D) and ethyl acetate (A) extracts of Wild-type and Commercial *S. fistulosus* by GC-MS. The compounds shown represent between 70 and 30% of the total compounds, respectively, as they only include compounds with a relative area greater than 10% in any of the extracts and a similarity index (MATCH) greater than 850.

### 2.7. Cytotoxic Activity

The extracts with the highest phytoconstituent content, and so the highest antioxidant activity, were chosen to evaluate their cytotoxic activity against the HT-29 and MCF-7 tumorigenic, and MCF-10 non-tumorigenic, cellular lines, as presented in Table 7.

The results presented in Table 7, show the viability of the HT-29 cancer cell line expressed as a percentage. It can be observed that only the Wild ethyl acetate extract, at a concentration of 100 µg·mL^−1^, exhibits significant differences compared to the negative control (C-), which represents the control without any extract. Consequently, the cell viability in the indicated treatment is lower than that of the control. Furthermore, at a concentration of 25 µg·mL^−1^, it is observed that significant differences increase compared to the negative control (C-). This suggests that at that concentration, the cancer cells could be using the extract as a source of nutrients, thereby promoting their proliferation.

The viability of the MCF-7 cancer cell line in relation to the two extracts showing the highest antioxidant activity is presented in Table 7. Notably, the ethyl acetate extract exposed in the Commercial sample with a concentration of 100 µg·mL^−1^ stands out, since it demonstrates the lowest percentage of cell viability. This indicates that fewer cells of this cancer type survived this treatment, with only 69.57% of cells remaining viable. 

When considering the cell viability of the non-cancerous cell line MCF-10, a remarkable finding is the cytotoxicity exhibited by the ethyl acetate extract of the Commercial *S. fistulosus* species, specifically, when exposed to a concentration of 100 µg·mL^−1^. The extract effectively reduced the viability of these cells by almost 50%, showing significant differences compared to both the negative control and the other extracts used in the study. On the other hand, the Wild-type extract proved to be harmless in both healthy and cancer cells.

## 3. Discussion

### 3.1. Phytoconstituents Found in S. fistulosus

In this study, it was observed that the concentration of phytoconstituents in the Wild-type sample increased significantly with increasing polarity and decreased when a high-polarity solvent, such as ethanol, was used. Consequently, dichloromethane and ethyl acetate (D and A), which are medium-polarity solvents, were identified as the most effective solvents for secondary metabolite extraction, which is also reflected in the Commercial sample. The choice of solvents for extraction plays a key role in determining both the quantity and quality of the extracts. The total content of secondary metabolites and their antioxidant capacity are highly dependent on the solvent used and the specific part of the plant used for extraction [32]. Solvents with similar polarity indices can effectively dissolve phytochemicals possessing similar or closely related polarity indices [32]. Therefore, it can be deduced that most of the phytoconstituents of *S. fistulosus* have medium polarity.

The highest concentration of phytoconstituents measured in Wild *S. fistulosus* was found to be phenols, followed by flavonoids, which is repeated in the Commercial species, while anthraquinones had the lowest concentration. It is important to note that the content of phenols with respect to the other phytoconstituents is considerably higher; this is because these compounds tend to play the role of defense against biotic and abiotic stress [33,34]. Furthermore, phenols are the most abundant group of phytoconstituents found in the plant kingdom [34]. In this study, the phenols/flavonoids ratio was 11 in the dichloromethane extract of the Wild species and 7.3 in the ethyl acetate extract of the Commercial species. Although the phytoconstituent content of *S. fistulosus* species has not been reported in the literature (Ruiz et al., 2019), other species of the same genus have been studied. Therefore, when comparing these findings with other studies on different species within the genus, it is evident that there is variability in the content of these phytoconstituents. For example, in a study on *S. hoggariensis*, a phenol/flavonoid ratio of 1.3 was obtained without anthraquinones [35] (see Table S1 in the Supplementary Material). In contrast, for *S. cineraria* species, only flavonoids were found, and no phenols or anthraquinones were detected [36] (see Table S1). 

In another study by Mohamed, AEHH., et al. (2022) on *S. glaucus*, only flavonoids were reported, while phenols and anthraquinones were not detected [37] (see Table S1). It is interesting to note that the content of phenols is significantly higher in *S. fistulosus* compared to other species of the same genus. The significance of these compounds lies in their antioxidant-associated anticancer properties, as well as their ability to mitigate the risk of cardiovascular and cognitive diseases [38].

Wild extracts of *S. fistulosus* show a higher content of phytoconstituents compared to Commercial samples. This may be attributed to the fact that the Wild species was exposed to significant concentrations of heavy metals. The presence of heavy metals has been reported to affect the metabolism of plant species, causing alterations in their physiological and metabolic functions. This, in turn, affects their photosynthetic capacity, due to a decrease in photosynthetic pigments, and may result in an increase in phytoconstituent content [39], and as shown in Table 2, the photosynthetic status of the Wild species is in good health. In other plants, including *Bacopa monnieri* (L.) Wettst. (Asteraceae), it has been found that the accumulation of an important phytoconstituent called bacoside can be influenced by metals, such as copper or cadmium. When the plant was tested at a copper concentration of 0.2 mM in the medium, there was an increase in bacoside production [40]. However, under abiotic stress conditions caused by cadmium, bacoside synthesis initially increases up to a certain threshold and then decreases due to the toxic effects of higher cadmium concentrations [40].

### 3.2. Antioxidant Activity of S. fistulosus Extracts 

It is important to emphasize that the extracts with the highest antioxidant activity are correlated with a higher content of phytoconstituents. Among *Senecio* species, Wild *S. fistulosus* extracts show a higher antioxidant activity compared to other studies within the same genus (see Table S1, Supplementary Material), and even with respect to the antioxidant activity of the Commercial species. Previous studies have shown that *S. hoggariensis* and *S. glaucus* have lower activity compared to *S. fistulosus*, as their IC_50_ values do not exceed 10 mg·mL^−1^ when assessing antioxidant activity using the DPPH assay [35,37] (see Table S1). Similarly, when compared to *S. cineraria* [36], *S. fistulosus* demonstrates a higher average inhibitory concentration (IC_50_ = 0.35 mg·mL^−1^, see Table S1, Supplementary Material). However, currently, none of these species are commercially exploited for their antioxidant properties, potentially due to consistently low amounts of bioactive phytoconstituents in the extracts. For instance, *S. fistulosus* exhibited 600 to 2000% less activity compared to the standard Trolox. Nevertheless, it is possible to enhance the qualitative and quantitative characteristics of plant extracts by employing a highly precise and accurate extraction protocol for the selective and effective extraction of phytochemicals from raw materials [41].

Regarding the total content of phenols, phytoconstituents, and DPPH antioxidant activity, it was observed that for extracts with medium polarity, the concentration of flavonoids and anthraquinones is responsible for the DPPH activity (see Table 4). The Wild species has a higher and significant content of phytoconstituents compared to the Commercial sample (see Table 2). Additionally, the Commercial sample showed a higher IC_50_ value than the Wild sample, which was double, indicating lower antioxidant activity (see Table 3). When measuring activities by FRAP or TRAP antioxidant assays, the stressed sample also showed lower antioxidant activity than the Wild sample, with a decrease of more than 50% (see Table 3), where the Spearman Rank correlation showed that this activity was due to the phenol content in the samples. These results show that the Wild species has higher and significant FRAP and TRAP activity compared to the Commercial sample.

### 3.3. Analysis of Phytoconstituents 

A comprehensive literature search was conducted to show the correlation between the compounds identified by GC-MS and the observed antioxidant and anticancer activities. Detailed information of studies reported in the literature on the compounds found can be found in Table S2 of the Supplementary Material. GC-MS analysis showed the presence of certain compounds in extracts D and A of the Wild sample that have been previously reported for their medicinal properties, which present a similar structure to that already reported by Ruiz-Vasquez et al. [30] (see Figure 1). These compounds include indolizine, a compound whose derivatives have been reported to show important antidiabetic, antimycoviral, anticancer, and other properties [42,43]. Phytol and its derivatives show a broad spectrum of biological activities, including anxiolytic, cytotoxic, metabolism-modulating, antioxidant, autophagy, apoptosis-inducing, antinociceptive, anti-inflammatory, immunomodulatory, and antimicrobial effects [44]. In the case of senecionine, it is a pyrrolizidine alkaloid with hepatotoxic and tumorigenic properties (see Table S2) [23]. However, it has been reported to possess anticancer activities [22,45,46]. It is highlighted that these compounds would be the most dominant in the Wild sample and, due to the characteristics, could justify the antioxidant or anticarcinogenic behavior of the Wild species. However, there are other compounds in lower concentrations, such as platyphylline, that have been reported to be toxic and even to promote certain diseases [47,48] (see Table S2). 

For the control Commercial, hexadecanoic acid (palmitic acid), found in both solvents D and A, according to the literature, presents properties that favor certain types of cancer and has been reported as responsible for antioxidant activity in the antioxidant activity of the *Senecio flammeus* species [49,50] (12.76 and 15.72%, respectively). Other compounds, such as (Z,Z,Z)-9,12,15-Octadecatrienoic acid, found in high concentrations in both extracts (linolenic acid: 18.36% for D and 19.57% for A) exhibit anticancer activity [51,52] (see Table S2), which could explain the cytotoxic activity against the MCF-7 and MCF-10 cell lines, which stand out with a viability percentage of 69.57 ± 5.31 and 51.91 ± 4.22, respectively, generating significant differences from the rest of the treatments, but demonstrating low selectivity of the extract.

### 3.4. Cytotoxic Activity of S. fistulosus Extract

Other plants of the same genus have already been described with anticancer properties. *Senecio leucanthemifolius* has demonstrated anticancer properties in the HeLa cell line using its essential oil, as reported by Ouchbani et al. [53]. In the case of *S. fistulosus* extracts, a cytotoxic effect was observed for the MCF-7 and the non-tumorigenic MCF-10 cell lines, particularly in Commercial species. However, no significant differences were found in the HT-29 cell line (see Table 7). Abiotic factors cause variations in the general phytochemical profiles of plants. Therefore, this variation could be attributed to the synthesis of distinct secondary metabolites with diverse pharmaceutical properties in response to different abiotic stress conditions [40]. Furthermore, it has been reported that the bioguided fractionation of the extract results in a significant enhancement of the cytotoxic effect [54]. (Z.Z.Z.)-9,12,15-octadecatrienoic acid was found by GC-MS analysis in the sample Commercial. The compound is known in the literature to inhibit cell proliferation in prostate, bladder, and breast cancer [51,52].

This study can serve as a starting point for future research on specific key metabolites of *Senecio fistulosus* with antioxidant and/or anticarcinogenic properties. The main aim would be to enhance the accumulation of bioactive secondary metabolites under different abiotic stress conditions. Additionally, various in vivo strategies can be explored and implemented to enhance the production of these important metabolites. The findings highlight the importance of considering abiotic stress conditions, such as metal contamination, when studying the medicinal properties of plants. However, it should be studied whether this plant is capable of accumulating metals, which could potentially pose health risks for people who consume it. Further research can build upon these results to explore strategies for maximizing bioactive compound accumulation and harnessing the therapeutic potential of *Senecio fistulosus* under different stress conditions.

## 4. Materials and Methods

### 4.1. Plants Collection and Extract Preparation 

Two specimens of *S. fistulosus*, namely, Wild and Commercial, were used in this study for their copper content in the aerial parts non-flowering, including stems and leaves. The Wild sample was located in soil with heavy metals content. The metal content in the topsoil of the sample under study showed the presence of arsenic (15.6 mg·kg^−1^), copper (284.7 mg·kg^−1^), and lead (16.9 mg·kg^−1^). The content of these elements was determined at Silob Chile Laboratory, by SM Method 23rd Edition 3030 K–3125 B; the results of this analysis are presented in the Supplementary Material. According to previous studies conducted in Puchuncavi valley, the elements in topsoil considered as part of anthropogenic contamination in the soils are As, Cd, Cu, and Pb, with concentrations ranging from 1.5 to 824, 0.01 to 28.4, 8 to 10,000, and 0.74 to 3389 mg·kg^−1^, respectively [28,55]. The species Wild was collected from the edge of Estuary Puchuncaví, located in the locality of Campiche in the commune of Puchuncaví (coordinates 32.73478° S–71.44666° W), in the region of Valparaíso, Chile, and was taxonomically identified by the curator of the herbarium VALPL of the Universidad de Playa Ancha, MSc. Pamela Ramirez (Voucher VALPL_PLV 2412). On the other hand, the Commercial sample was obtained from an alternative medicine store, where it is sold as dried leaves and stems.

The extract of plant materials (Wild and Commercial) was obtained from 300 g dried leaves and stems under dark and room temperature conditions at the Laboratory of Natural Products and Organic Synthesis (LPNSO), Department of Geography and Natural Sciences, Universidad de Playa Ancha, Valparaíso, Chile. Plant extracts with different organic solvents were obtained using ultrasound equipment (Elma TI-H-5), with two impacts of 1 h each, separated by 24 h. Organic solvents were used in increasing polarity (hexane (H), dichloromethane (D), ethyl acetate (A), and ethanol (E)). The percentage yield has been obtained by Equation (1). Extract concentration was performed in a rotary evaporator (LabTech EV400H) with a vacuum pump (LabTech VP30) at 45 °C for all solvents. These extracts were used to measure phytoconstituent content, antioxidant activity, and cytotoxic activity. For subsequent experiments, the samples were persevered at −4 °C. 

The percentage yield (%R) is calculated according to the following equation:

(1) $$\%R=\frac{Extract}{Dry\;mass}\times 100$$

### 4.2. Photosynthetic Capacity 

The photosynthetic capacity of *S. fistulosus* was determined only in the Wild species to assess its health status, where the effective quantum yield (Yield II) was measured at an irradiance of 1970 µmol photons m^−2^ s^−1^ at 10:00 a.m. Furthermore, the maximum yield, the maximum potential of the electron transport chain (*F_v_/F_m_*) obtained after 15 min of shade, was observed [56]. 

The in situ electron transport rate (ETR in situ) that determines the in situ productivity in *S. fistulosus* under the aforementioned conditions is reported (Equation (2)).

(2) $$ETR\;in\;situ=E\times Yield\left(II\right)\times Absortance\times PSII$$

where E is an Irradiance; Yield (II) is photosynthetic capacity in situ; the absorbance value, which refers to the amount of light that passes through photosystem II two, was recorded; and PSII in vascular plants is 0.5.

### 4.3. Phytoconstituents Determination 

The phenol, flavonoid, and anthraquinone components of *S. fistulosus* different extracts were quantitatively identified by UV–Visible spectrophotometric methods. The content of phytoconstituents and antioxidant activity was measured for extracts with different solvents from the aerial parts of *S. fistulosus*.

The total phenolic content of the aerial part of the species under study were using the Folin–Ciocalteu colorimetric assay [57]. Briefly, 0.5 mL of the extract (1 mg·mL^−1^ of the dry extract) was added to 2.5 mL of Folin–Ciocalteu reagent (0.2 N), and the solution was mixed thoroughly. After 5 min, 2 mL of a 7.5% sodium carbonate (Na_2_CO_3_) aqueous solution was added, and the mixture was allowed to stand in the dark for 2 h at room temperature. The absorbance at 700 nm was measured using a UV–visible spectrophotometer (Rayleigh UV-2601). Gallic acid was used as a standard to establish the calibration curve, and the total phenolic content was calculated and expressed as mg of gallic acid equivalents (GAE) per g of dry extract. All samples were worked in triplicate. 

The total flavonoid content in the aerial part of *S. fistulosus* extract was determined using the aluminum chloride colorimetric method [57,58]. In brief, 500 mL of extract solution (1.0 mg·mL^−1^ of dry extract) was added to a 2% solution of aluminum chloride in ethanol. After 10 min, the absorbance at 415 nm was measured using a UV–visible spectrophotometer (Rayleigh UV-2601) and a standard solution as against the reagent blank, which consists of 500 mL of the extract with 500 mL of ethanol without AlCl_3_. A standard calibration curve was generated using known concentrations of a freshly prepared quercetin solution. Therefore, the total flavonoid concentration in the extract sample was calculated and expressed as mg quercetin equivalents (QEs) per g of dry extract. The assay was carried out in triplicate. 

The total anthraquinone content in the aerial part of *S. fistulosus* extract was determined using the aluminum chloride colorimetric method [57], In brief, 500 mL of extract solution (1.0 mg·mL^−1^ of dry extract) was added to an equal amount of aluminum chloride solution in 2% ethanol. After 10 min, the absorbance at 485 nm was measured using a UV–visible spectrophotometer (Rayleigh UV-2601) against the reagent blank, which consists of 500 mL of the extract with 500 mL of ethanol without AlCl_3_. A standard calibration curve was generated using known concentrations of emodin. Therefore, the total anthraquinone content was calculated and expressed in mg emodin equivalents (EEs) per g dry extract. The estimation of total anthraquinone content was carried out in triplicate. 

### 4.4. Determination of Antioxidant Activity

Different plant extracts obtained were evaluated for their antioxidative activity by three methodologies, 2,2′-diphenyl-1-picrylhydrazyl stable free radical scavenging activity (DPPH∙), ferric reducing power analysis (FRAP), and total reactivity antioxidant potential (TRAP), which are UV–Visible spectrophotometric methods that seek to determine such activity in the extracts under study. 

The DPPH∙ free radical scavenging activity was conducted following the method outlined in the current literature [57,59]. First, 100 µL of each extract sample (at concentrations of 1.0 mg·mL^−1^, 3.0 mg·mL^−1^, 5.0 mg·mL^−1^, 7.0 mg·mL^−1^, and 10.0 mg·mL^−1^) was added to 2.9 mL of DPPH solution (concentration of 50 µM) and incubated at room temperature for 15 min. The absorbance at 517 nm was measured spectrophotometrically against a blank sample using the Rayleigh UV-2601 spectrophotometer. The absorbance (Abs.) was measured at zero minutes and after 15 min of incubation with 100 µL of the sample and 2.9 mL of ethanol. All experiments were performed with gallic acid and Trolox^TM^ used as a positive control. The scavenging activity was determined by calculating the percentage inhibition of the DPPH∙ radical from which the IC_50_ value was obtained and reported. All measurements were performed in triplicate.

The ferric reducing power was determined following the methodology outlined in the current literature [57,60]. FRAP reagent was prepared by adding 300 mM Buffer solution, 10 mM 2,4,6-tri(2-pyridyl)-s-triacin (TPTZ), and 20 mM ferric chloride, at a ratio of 10:1:1, respectively. Briefly, 100 µL of the sample (which should be at a 1 gr/mL concentration) was added to 3 mL of FRAP reagent and 300 µL of distilled water. The solution was mixed thoroughly and incubated for 30 min. The absorbance at 593 nm was measured spectrophotometrically using the Rayleigh UV-2601 spectrophotometer. Distilled water was blank. A standard calibration curve was generated using known concentrations of Trolox^TM^ (0–120 mg·mL^−1^). The reducing capacity of the extracts was expressed in mM TEAC. All measurements were replicated three times.

The TRAP assay was following the method described in previous studies [61]. The aim was to evaluate the antioxidant activity of secondary metabolites employing spectrophotometric methods. First, 150 µM of ABTS∙ 2,2’-azino-bis (3-ethylbenzothialin-6-sulfonic acid) (ABTS) and 10 mM of 2,2’-azo-bis (2-amidino propane) (ABAP) were mixed at a 1:1 ratio and incubated with constant stirring at 45 °C for 30 min, followed by cooling. The absorbance at 734 nm was measured spectrophotometrically using the Rayleigh UV-2601 spectrophotometer, every 10 s until 50 s, against a blank solution corresponding to ABTS (the sample contained 1 mL of the mixture and 10 µL of the extract, which were at a 1:100 ratio). A standard calibration curve was generated using known concentrations of Trolox^TM^ (0–120 mg·mL^−1^). The results are expressed in mM Trolox^TM^ equivalent antioxidant capacity (TEAC μM), using a Trolox^TM^ standard curve (from 0–120 mg·L^−1^). All measurements were repeated three times.

### 4.5. Cytotoxicity Activity

For the evaluation of the viability of HT-29 and MCF-7 cancer cells and non-tumorigenic line MCF-10, obtained from the Bioassays laboratory of the University of Valparaiso, provided by Dr. Joan Villena, they were treated with the extracts with the highest content of phytoconstituents and antioxidant activity of *S. fistulosus*, that is, extracts A and D of both treatments. The sulforhodamine B assay is carried out as expressed in the current literature [62,63,64].

The extracts of *S. fistulosus* should be kept protected from light and should be worked fresh, and the mother solution should be kept at −20 °C and the working solution at 4 °C. Maintaining these conditions, they should be diluted in dimethyl sulfoxide (DMSO) or ethanol to a concentration of 0.1 M, which will be the stock solution; with this, a concentration of 1 mM will be obtained, which will be the working concentration, from which four different concentrations were made (500 µg·mL^−1^, 250 µg·mL^−1^, 125 µg·mL^−1^, 62.5 µg·mL^−1^). This should be followed by filtration. 

The cell lines used should be washed and trypsinized before reaching 90–100% confluence, a homogeneous 1:1 solution (cell suspension: 0.4% (wt/vol) trypan blue) should be achieved, and the seeding cell density should be at 3 to 5 × 10^3^ cells/well. The cells are incubated at 37 °C, in a 5% CO_2_ and 95% O_2_ atmosphere incubator, and treated with the extracts prepared above for 72 h. The cells are then fixed with trichloroacetic acid for 1 h at 4 °C, washed by immersion, stained with 0.1% sulforhodamine B dissolved in acetic acid for 30 min, and washed 3 times, but this time with 1% acetic acid. Finally, the cell density is analyzed in a plate reader at 540 nm, and the viability of the cells under study will be estimated based on the percentage of inhibition. All measurements are in triplicate.

### 4.6. Analysis of Phytoconstituents by GC-MS Technique

Only the most active extracts (D and A from Wild-type and Commercial species) were analyzed by GC-MS (TRACE 1610, ISQ 7610 mass detector. EEUU). Extracts (1.0 μL) were injected in spitless mode (5 min) on an RTX-5MS column (30 m, 0.25 mm diameter, 5% diphenyl and 95% dimethylpolysiloxane), with helium as the carrier gas at a constant flow rate of 1.5 mL·min^−1^ and a column pressure of 92.3 KPa. The injector temperature was 230 °C. The thermal profile was as follows: the temperature was held for 3 min at 50 °C and then increased at a rate of 25 °C·min^−1^ to 250 °C, and then that temperature was held for 15 min.

The mass sweep was operated in electron impact ionization (70 eV), scanning from the m/s range of 30–600 in the full scan mode. The mass spectra were compared with the internal spectra database. Compounds in the chromatograms were identified by comparing their mass spectra with those in the NIST/EPA/NIH mass spectral library [65]. Chromatographic peaks were considered “unknown” when their similarity index (MATCH) and inverse similarity index (RMATCH) were less than 850, and were discarded [66]. These parameters refer to the matching ability of the target spectrum to the NIST MS Search 2.4 Library standard spectrum (a value of 1000 indicates a perfect fit).

### 4.7. Statistical Analysis

Data are reported, using the STATISTICA 7.0 program (StatSoft, Hamburg, Germany), as mean values ± standard deviation (SD). A distribution test was performed to identify the nature of the data obtained from the spectrophotometric tests (phytochemical profile, antioxidant activity, and anticarcinogenic activity). The results of this test for the phenol and flavonoid extracts were found to be parametric, for which they were subjected to an ANOVA test; the rest of the tests were found to be nonparametric, for which the Kruskal–Wallis test was performed for multiple independent variables. In all these cases, the confidence level is 95%. The tests presented a *p* value < 0.05; therefore, Tukey’s tests were performed. On the other hand, the correlation Spearman Rank Order Correlations between phytoconstituents and antioxidant and anticarcinogenic activity was determined.

## 5. Conclusions

The main phytoconstituents found in the extracts of the aerial parts of Wild and Commercial *S. fistulosus* demonstrate a medium polarity. Phenols, followed by flavonoids, are the most abundant phytoconstituents measured, according to UV–Visible spectrophotometric methods, in these extracts. The antioxidant activity of *S. fistulosus* under abiotic stress exhibits a higher value compared to that reported in other studies involving species from the same genus, and even compared to the Commercial control. The abiotic stress in this study resulted in an increase in the antioxidant activity of *S. fistulosus*, which is likely the cause of the observed increase in its phytoconstituent content. In the case of the MCF-7 tumorigenic cell line and MCF-10 non-tumorigenic cell line, a cytotoxic effect was observed of *S. fistulosus* extracts of Commercial species. However, no significant differences were found in the HT-29 cell line. According to GC-MS analysis, molecules such as hexadecanoic acid and (Z,Z,Z)-9,12,15-octadecatrienoic acid could be associated with this cytotoxic activity observed in the Commercial sample.

In conclusion, our study suggests that extracts derived from the aerial parts of *S. fistulosus* under abiotic stress possess the potential to demonstrate antioxidant activity, over the Commercial control. Therefore, it is important to consider that industrial pollution in the Puchuncaví Valley may have a positive impact on its antioxidant properties, while potentially decreasing its cytotoxic activities, being innocuous for the cancer cell lines and the healthy control used in this study.

## Figures and Tables

**Table 1:** Percentage yield of extracts of aerial part of *S. fistulosus* for Wild and Commercial.

Percentage Yield (%)		
Extracts	Wild	Commercial
H	1.61	0.85
D	0.81	0.98
A	0.75	0.35
E	9.24	2.26

Hexane (H), dichloromethane (D), ethyl acetate (A), and ethanol (E).

**Table 2:** Photosynthetic capacity of the aerial part of the species Wild *S. fistulosus*.

Yield II	*F_v_/F_m_*	ETR In Situ (µmol m^−2^ s^−1^)	Absorptance	PAR (µmol photons m^−2^ s^−1^)
0.46 ± 0.04	0.79 ± 0.02	439.83 ± 35	0.97 ± 0.02	1970

Yield II corresponds to the photosynthetic capacity in situ; *F_v_/F_m_* corresponds to the maximum potential of the electron transport chain; ETR in situ corresponds to the value of the electron transport rate; Absorptance is the amount of light passing through photosystem 2 in the electron transport chain; and PAR corresponds to irradiance in situ.

**Table 3:** Content of secondary metabolites in different extracts of aerial part of *S. fistulosus* for Wild and Commercial species obtained in the UV-Vis spectrophotometric assays.

Extracts	Phenols (mg·L^−1^ AGE)		Flavonoids (mg·L^−1^ QE)		Anthraquinones (mg·L^−1^ EE)	
	Wild	Commercial	Wild	Commercial	Wild	Commercial
H	70.63 ± 1.05 ^a^	37.57 ± 1.02 ^e^	4.17 ± 0.19 ^a^	1.08 ± 0.48 ^d^	0 ± 0.00 ^a^	0 ± 0.00 ^d^
D	169.48 ± 1.64 ^b^	51.55 ± 0.43 ^f^	15.42 ± 0.97 ^b^	12.74 ± 1.66 ^e^	31.08 ± 1.29 ^b^	3.60 ± 0.70 ^de^
A	160.39 ± 1.29 ^c^	95.39 ± 1.22 ^g^	9.15 ± 0.48 ^c^	13.10 ± 0.82 ^e^	13.34 ± 2.04 ^c^	9.63 ± 1.46 ^e^
E	27.53 ± 1.84 ^d^	40.03 ± 1.12 ^e^	4.70 ± 1.26 ^a^	2.66 ± 0.42 ^ad^	0.55 ± 0.42 ^d^	0.69 ± 0.21 ^d^

Hexane (H), dichloromethane (D), ethyl acetate (A), and ethanol (E). Superscript letters (a to g) of the same assay represent significant differences, with *p* < 0.05.

**Table 4:** Antioxidant activity of the aerial part of different extracts for *S. fistulosus*.

Extracts	DPPH (IC_50_)		FRAP (TEAC mM)		TRAP (TEAC mM)	
	Wild	Commercial	Wild	Commercial	Wild	Commercial
H	7.18 ± 0.19 ^a^	7.49 ± 0,09 ^a^	0.24 ± 0.01 ^a^	0.08 ± 0.01 ^c^	0.006 ± 0.00056 ^a^	0.0057 ± 0.0002 ^ad^
D	1.89 ± 0.01 ^b^	6.34 ± 0.17 ^d^	0.6 ± 0.04 ^b^	0.09 ± 0.03 ^c^	0.016 ± 0.00052 ^b^	0.0042 ± 0.0005 ^d^
A	1.99 ± 0.01 ^b^	4.24 ± 0.07 ^e^	0.54 ± 0.02 ^b^	0.17 ± 0.01 ^c^	0.016 ± 0.00010 ^c^	0.0084 ± 0.0002 ^e^
E	3.29 ± 0.04 ^c^	9.2 ± 0.28 ^f^	0.12 ± 0.01 ^c^	0.09 ± 0.01 ^c^	0.016 ± 0.00007 ^d^	0.0032 ± 0.0001 ^f^
TROLOX	0.26 ± 0.00 ^g^	0.26 ± 0.00 ^g^	n.a.	n.a.	n.a.	n.a.
Gallic acid	0.06 ± 0.00 ^g^	0.06 ± 0.00 ^g^	n.a.	n.a.	n.a.	n.a.

Hexane (H), dichloromethane (D), ethyl acetate (A), and ethanol (E). Letters in superscript (a to g) of the same assay represent significant differences, with *p* < 0.05.

**Table 5:** Spearman Rank Order Correlations for the content of secondary metabolites and antioxidant activity.

	DPPH	FRAP	TRAP
PHE	−0.586957	0.811304	0.778261
FLA	−0.726957	0.571304	0.448696
ANT	−0.725217	0.519130	0.549565

phenols (PHE), flavonoids (FLA), and anthraquinones (ANT). Significant differences (*p* < 0.05).

**Table 6:** Gas chromatography of dichloromethane and ethyl acetate extracts of Wild *S. fistulosus* and the Commercial standard.

Extract of Wild *S. fistulosus* in Dichloromethane								
Ret. Time min	Peak Name	Class	Rel. Area%	SI	RSI	MF	MW (g/mol)	CAS
5.514	2-Pentanone, 4-hydroxy-4-methyl-	Diacetone Alcohol	13.71	939	939	C_6_H_12_O_2_	116.16	123-42-2
7.871	Indolizine	Aromatic heterocycle isomer, nucleus of various alkaloids	12.18	893	897	C_8_H_7_N	117.15	274-40-8
11.918	Neophytadiene	Sesquiterpenoids	20.23	884	914	C_20_H_28_	278.5	504-96.1
16.564	Senecionine	Pyrrolizidine alkaloid	40.02	867	868	C_18_H_25_NO_5_	335.4	1130-01-8
	Unknown	-	13.86					
Total known compounds			86.14					
**Extract of Wild *S. fistulosus* in Ethyl Acetate**								
**Ret. Time min**	**Peak Name**		**Rel. Area%**	**SI**	**RSI**	**MF**	**MW (g/mol)**	CAS *
5.517	2-Pentanone. 4-hydroxy-4-methyl-	Diacetone Alcohol	11.60	927	927	C_6_H_12_O_2_	116.16	123-42-2
11.918	Neophytadiene	Sesquiterpenoids	20.23	888	927	C_20_H_28_	278.5	504-961
13.592	Phytol	Acyclic diterpenoids	42.79	913	913	C_20_H_40_O	296.5	150-86-7
16.564	Senecionine	yrrolizidine alkaloid	15.43	849	850	C_18_H_25_NO_5_	335.4	130-01-8
	Unknown	-	19.25					
Total known compounds			80.75					
**Extract of *S. fistulosus* Commercial Control in Dichloromethane**								
**Ret. Time min**	**Peak Name**		**Rel. Area%**	**SI**	**RSI**	**MF**	**MW (g/mol)**	CAS *
12.548	n-Hexadecanoic acid	Long-chain fatty acids	12.76	857	869	C_16_H_32_O_2_	256.42	57-10-3
13.799	(Z,Z,Z) 9,12,15-Octadecatrienoic acid	Organic acid	18.36	910	910	C_18_H_30_O_2_	278.4	453-40-1
	Unknown	-	68.88					
Total known compounds			31.12					
**Extract of *S. fistulosus* Commercial Control in Ethyl Acetate**								
**Ret. Time min**	**Peak Name**		**Rel. Area%**	**SI**	**RSI**	**MF**	**MW (g/mol)**	**CAS**
12.548	n-Hexadecanoic acid	Long-chain fatty acids	19.57	861	869	C_16_H_32_O_2_	256.42	57-10-3
13.799	(Z.Z.Z)-9.12.15-Octadecatrienoic acid	Organic acid	18.36	905	905	C_18_H_30_O_2_	278.4	463-40-1
	Unknown	-	62.07					
Total known compounds			37.93					

Where SI corresponds to the similarity index, RSI corresponds to the inverse similarity index, MF corresponds to the molecular formula, and MW corresponds to the molecular weight of the compound identified by GC-MS. (*) CAS corresponds to the numerical designation assigned to chemicals by the US Chemical Abstracts Service.

**Table 7:** Cytotoxicity of extracts of the aerial part of the species *S. fistulosus*, on the HT-29 cell line, MCF-7 cell line, and MCF-10 cell line.

Percentage of Viability of the HT-29 Cell Line (%)				
Extracts	Dichloromethane		Ethyl Acetate	
Treatment	Wild	Commercial	Wild	Commercial
C-	100.48 ± 1.17	100.48 ± 1.17	100.48 ± 1.17	100.48 ± 1.17
100 µg/mL	95.25 ± 6.27	96.13 ± 7.60	93.92 ± 3.99 ^b^	95.24 ± 8.26
50 µg/mL	100.46 ± 3.00	103.98 ± 9.49	98.50 ± 3.72	102.51 ± 11.19
25 µg/mL	102.36 ± 5.74 ^a^	103.45 ± 5.96	94.65 ± 3.83	95.55 ± 4.60
12.5 µg/mL	101.45 ± 4.24	105.26 ± 6.64	99.48 ± 7.07	103.48 ± 5.61
**Percentage of Viability of the MCF-7 Cell Line (%)**				
**Extracts**	**Dichloromethane**		**Ethyl Acetate**	
**Treatment**	**Wild**	**Commercial**	**Wild**	**Commercial**
C-	106.11 ± 10.21	106.11 ± 10.21	106.11 ± 10.21	106.11 ± 10.21
100 µg/mL	93.09 ± 7.85 ^c^	88.80 ± 9.95 ^d^	97.69 ± 2.71	69.57 ± 5.31 ^e^
50 µg/mL	104.66 ± 5.65	105.00 ± 10.08	102.22 ± 3.65	97.65 ± 11.66
25 µg/mL	105.41 ± 5.24	106.19 ± 10.56	102.47 ± 5.90	98.65 ± 9.02
12.5 µg/mL	108.63 ± 6.66	104.27 ± 5.44	107.29 ± 5.98 ^d^	105.29 ± 6.95
**Percentage of Viability of the MCF-10 Cell Line (%)**				
**Extracts**	**Dichloromethane**		**Ethyl Acetate**	
**Treatment**	**Wild**	**Commercial**	**Wild**	**Commercial**
C-	109.91 ± 14.16	109.91 ± 14.16	109.91 ± 14.16	109.91 ± 14.16
100 µg/mL	93.95 ± 5.55 ^f^	73.27 ± 7.96 ^h^	98.01 ± 5.74 ^j^	51.91 ± 4.22 ^k^
50 µg/mL	124.18 ± 8.40	109.20 ± 9.14	101.34 ± 5.17	95.07 ± 10.60 ^l^
25 µg/mL	128.50 ± 14.24 ^g^	125.44 ± 6.39 ^i^	104.87 ± 11.34	108.97 ± 9.05
12.5 µg/mL	134.65 ± 8.51 ^g^	120.70 ± 9.73	126.27 ± 8.91	129.91 ± 9.50 ^m^

Letters in superscript (a to m) of the same assay represent significant differences (*p* < 0.05), with respect to the control without extract (C-).

## Data Availability

The data presented in this study were obtained through laboratory analyses and they were not available in public databases.

## Supplementary Materials

The following supporting information can be downloaded at: https://www.mdpi.com/article/10.3390/molecules28207038/s1, Table S1: Studies reported in literature on extraction of secondary metabolites; Table S2: Studies reported in the literature on compounds found in the GC–MS technique; Figure S1: GC-MS chromatograms of the dichloromethane extract of the aerial part of Wild *S. fistulosus*; Figure S2: GC-MS chromatograms of the ethyl acetate extract of the aerial part of Wild *S. fistulosus*; Figure S3: GC-MS chromatograms of the dichloromethane extract of the aerial part of Commercial *S. fistulosus*; Figure S4: GC-MS chromatograms of the ethyl acetate extract of the aerial part of Commercial *S. fistulosus*.
