# Supplementary material for: Effect of Industrial Pollution in Puchuncaví Valley on the Medicinal Properties of Senecio fistulosus Poepp. ex Les (Asteraceae): Content of Phytoconstituents and Their Antioxidant and Cytotoxic Activities
Source: Molecules. 2023 Oct 12;28(20):7038. doi: 10.3390/molecules28207038 (PMC10609587; doi:10.3390/molecules28207038)

Supplementary Materials:

Table S1. Studies reported in literature on extraction of secondary metabolites.

Specie	Phe.	Flav.	Ant. (mg/EE)	DPPH (mg/mL) IC_{50}	FRAP (TEACmM)	TRAP (TEACmM)	Solvent	Reference
S. fistulosus wild	169.48 ± 1.64 mg/L GAE	15.42 ± 0.97 mg/L QE	31.08 ± 1.29	1.89 ± 0.01	0.6 ± 0.04	0.016 ± 0.00	Dichloromethane	This study
S. fistulosus Commercial	95.39 ± 1.22 mg/L GAE	13.10 ± 0.82 mg/L QE	9.63 ± 1.46	4.24 ± 0.07	0.54 ± 0.02	0.016 ± 0.00	Ethyl acetate	This study
S. hoggariensis	100.27 ± 0.74 µg GAE/mg	80.06 ± 1.14 µg QE/mg	-	46.40 ± 3.95 (µg /mL)	-	-	Ethyl acetate extract	Arab, Y., et al., 2022
S. cineraria	-	60.16 mg/g EQ	-	0.35 mg/mL	-	-	Polar extract	Ababsa, ZA., et al., 2018
S. glaucus	O,11 µg/g GAE	1,17 µg/g QE	-	10 µg by 30.42%	-	-	Hexane-Dichloromethane (1:1) extract	Mohamed, AEHH., et al., (2022)

Table S2. Studies reported in the literature on compounds found in the GC–MS technique.

Secondary metabolite	Type	Observation	Reference
Indolizine	Alkaloid	Pharmaceutical products with significant anti-inflammatory, analgesic, antimicrobial, antiexudative, antidiabetic, antimicoviral, anticancer, hypoglycemic and other activities.	Monreal-Corona et al., 2023; Flitsch *et al*., 1984; Salem *et al*., 2018; Harma, V., & Kumar, V. 2014; Basavaraj, M., *et al*., 2022
Germacrene D	Sesquiterpeno	Antimicrobial and insecticidal properties, although they also play a role as insect pheromones.	Li et al., 2021
Neophytadiene	Diterpene	Antimicrobial properties	Cáceres et al., 2015
Phytol	isoprenoid alcohol	Its derivatives present a wide range of biological activities such as, anxiolytic, cytotoxic, metabolism modulating, antioxidant, autophagy and apoptosis inducing, antinociceptive, anti-inflammatory, immunomodulatory and antimicrobial effects.	Ding *et al*., 2021; Gutbrod *et al*., 2021; Sanjeev *et al*., 2020; Islam, MT., *et al*., 2018; Islam, MT., *et al*., 2018
Senecionine	Alkaloid	Is one of the most studied hepatotoxic and carcinogenic	Xia *et al*., 2020; Blanco, C., *et al*., 2020; Monreal-Corona, *et al*., 2023; Sanjeev *et al*., 2020
Platyphylline	Alkaloid	Defense mechanism against herbivores.	Alves Paiva 2004; Ebmeyer 2019
Hexadecanoic acid	Saturated free fatty acid	Positive effects on melanoma metastasis in mice.	Lv *et al*., 2023
9,12,15-Octadecatrienoic acid. (Z,Z,Z)-	Linolenic Acid	It possesses beneficial in diabetes, cancer and heart disease, in addition to antifungal activity.	Javaid *et al*., 2023; Prasanna 2017
1-Heptatriacotanol	alcoholic compound	Could prevent cancer development	Oluwasina *et al*., 2023

Figure S1. GC-MS chromatograms of the dichloromethane extract of the aerial part of Wild *S. fistulosus*.

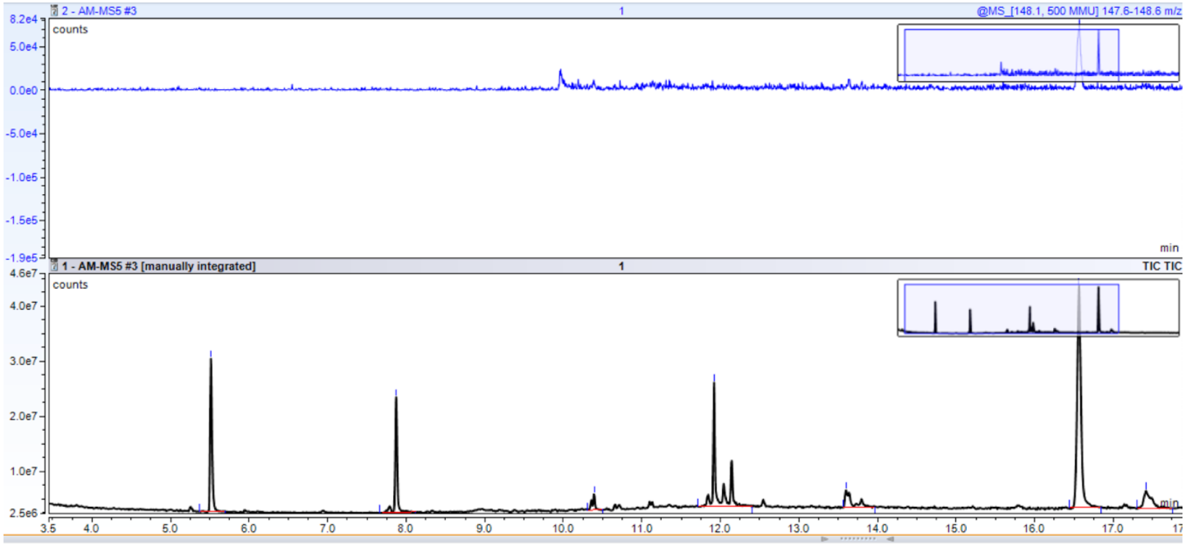

Figure S2. GC-MS chromatograms of the ethyl acetate extract of the aerial part of Wild *S. fistulosus*.

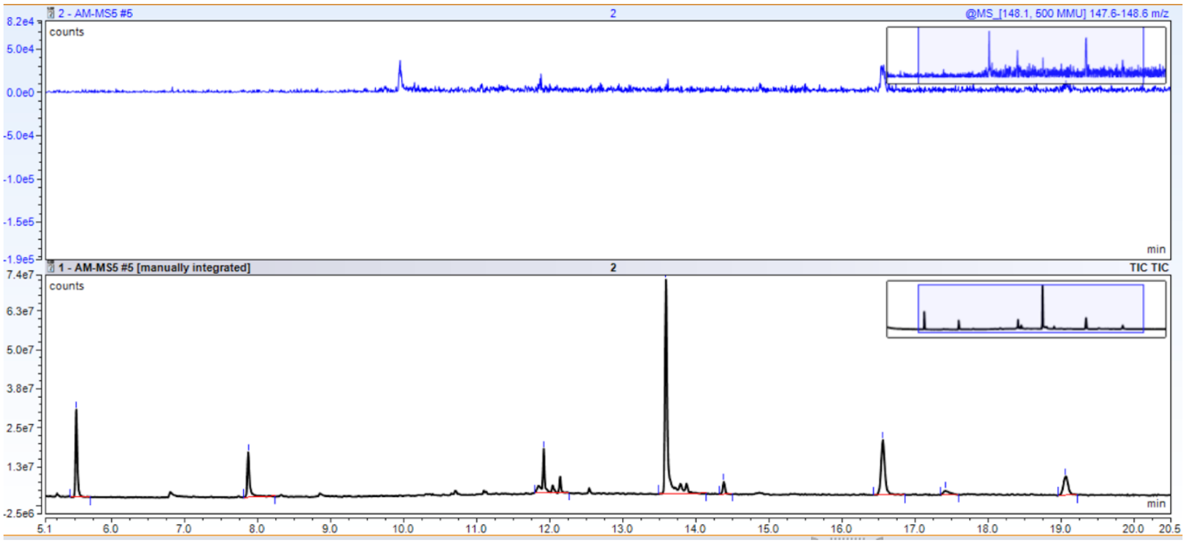

Figure S3. GC-MS chromatograms of the dichloromethane extract of the aerial part of Commercial *S. fistulosus*.

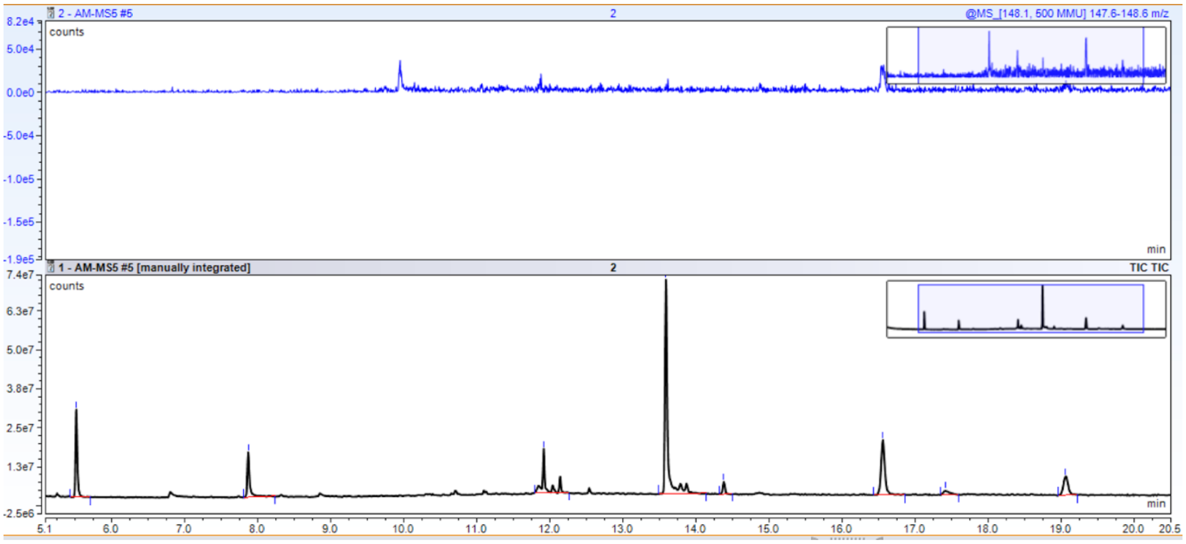

Figure S4. GC-MS chromatograms of the ethyl acetate extract of the aerial part of Commercial *S. fistulosus*.

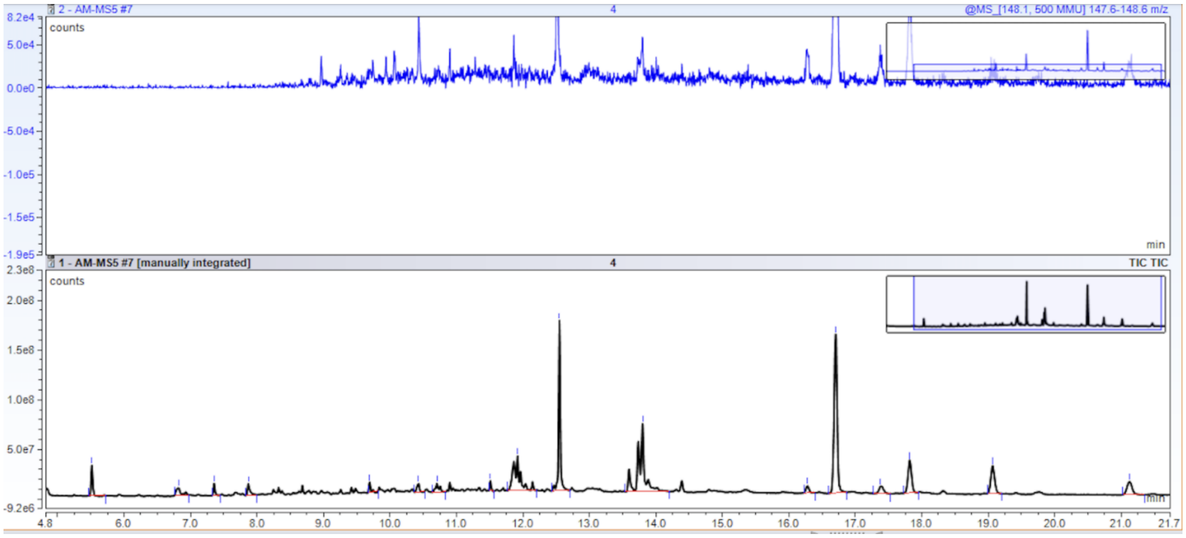

Supplement: Supplementary file 1 [file molecules-28-07038-s001.zip › molecules-2596575-supplementary.pdf]
